# Infestation of an endemic arbovirus area by sympatric populations of *Aedes aegypti* and *Aedes albopictus* in Brazil

**DOI:** 10.1590/0074-02760190437

**Published:** 2020-05-18

**Authors:** Rosângela Maria Rodrigues Barbosa, Maria Alice Varjal de Melo-Santos, José Constantino Silveira, Maria Helena Neves Lobo Silva-Filha, Wayner Vieira Souza, Cláudia Maria Fontes de Oliveira, Constância Flávia Junqueira Ayres, Morgana do Nascimento Xavier, Marina Praxedes Rodrigues, Suzane Alves dos Santos, Mitsue Maia Nakazawa, Lêda Narcisa Regis

**Affiliations:** 1 Fundação Oswaldo Cruz-Fiocruz, Instituto Aggeu Magalhães, Departamento de Entomologia, Recife, PE, Brasil; 2 Fundação Oswaldo Cruz-Fiocruz, Instituto Aggeu Magalhães, Departamento de Saúde Coletiva, Recife, PE, Brasil

**Keywords:** vectors, arboviruses, egg monitoring, surveillance, ovitraps, dengue

## Abstract

**BACKGROUND:**

*Aedes aegypti* and *Aedes albopictus* are the most important arbovirus vectors in the world.

**OBJECTIVES:**

This study aimed to investigate and compare the infestation pattern of these species in a neighbourhood of Recife, Brazil, endemic for arboviruses in 2005 (T1) and 2013 (T2).

**METHODS:**

Infestation, distribution and relative abundance of these sympatric species were recorded by egg collection using a network of 59 sentinel ovitraps (s-ovt) at fixed sampling stations for 12 months in T1 and T2.

**FINDINGS:**

A permanent occupation pattern was detected which was characterised by the presence of egg-laying females of one or both species with a high ovitrap positivity index (94.3 to 100%) throughout both years analysed. In terms of abundance, the total of eggs collected was lower (p < 0.005) in T2 (146,153) than in T1 (281,103), although ovitraps still displayed a high index of positivity. The spatial distribution showed the presence of both species in 65.1% of the 148 s-ovt assessed, while a smaller number of traps exclusively contained *Ae. aegypti* (22%) or *Ae. albopictus* (13.2%) eggs.

**MAIN CONCLUSIONS:**

Our comparative analysis demonstrated the robustness of the spatial occupation and permanence of *Ae. aegypti* and *Ae. albopictus* populations in this endemic urban area.

The incidence of dengue cases has grown worldwide in the past fifty years, and dengue is now considered the most significant arboviral disease transmitted to humans. It has been estimated that 390 million new infections occur annually in tropical and subtropical regions of the world.^1^ Despite global control efforts, the expansion of the territories occupied by established populations of *Aedes aegypti* and *Aedes albopictus* have been reporded.^2^ This has been associated with an increase in the scale and severity of dengue epidemics and the emergence and the rapid spread of other arboviruses (e.g., Chikungunya, Yellow Fever and Zika) that can be carried by these mosquitoes.^3^ In the Americas, *Ae. aegypti* is considered the primary vector of the dengue (DENV), Chikungunya (CHIKV), Yellow Fever (YFV) and Zika (ZIKV).^4^ In Europe, dengue cases have been associated with *Ae. albopictus* along with Chikungunya outbreaks such as the one recorded in 2007 in Ravenna, Italy, with over 200 confirmed cases and one death.^5^ *Ae. albopictus* is present in at least 24 of 27 Brazilian states.^6^ In Brazil evidence of the vertical transmission of the dengue virus (DENV) in populations of *Aedes albopictus* kept in the laboratory and in samples collected in the field have recorded.^7^

Dengue was reintroduced in Brazil in 1981-1982 and was associated to serotypes DENV-1 and DENV-4.^8^ A wide-ranging National Program for Dengue Control (PNCD) was established in 1996 and currently operates in more than 5,000 Brazilian municipalities.^9^ The PNCD actions for monitoring and controlling the vector are based on the estimated number of infested houses using a visual search for larvae-pupae in household breeding grounds, bimonthly applications of larvicides throughout the year in water containers, campaigns to eliminate larval breeding sources and sporadic focal adulticide spraying in cases of active viral transmission.^10^ Within the last decades, the country experienced four major epidemics, associated with the predominant viral serotype DENV-1, DENV-3, DENV-2, and DENV-4 in 1998, 2002, 2008 and 2010, respectively.^11^ In the past years, dengue epidemics have been caused by the circulation of more than one serotype. In 2015, states from the northeast of Brazil reported the first outbreaks of ZIKV and CHIKV infections.^12^ One of the major challenges for the surveillance and control of *Aedes* species in the scope of PNCD is the limitation of the index employed to perform the surveillance of this species in urban areas.^13^ Those are based on the percentage of houses infested with larvae and/or pupae (House index) and the number of containers/reservoirs found positive for them per a set of 100 houses inspected (Breteau index).^10^ However, these metrics have shown low levels of infestation or even negative results in areas where *Ae. aegypti* is present in 60% to 90% of the surveyed houses.^13^ A more sensitive method for the large-scale sampling of *Aedes* species must be adopted to evaluate the abundance and impact of vector control strategies.

In fact, the complexity of the contemporary urban environments suggests the need to develop new surveillance tools to replace the current methods, which date back to 80 years ago. A tool commonly used to monitor and control *Aedes* is the oviposition traps or ovitraps,^14^ which have been shown to be more sensitive than searching for larvae and/or pupae that can be spread in a wide range of cryptic breeding sites. In recent years, various traps for capturing *Aedes* adults and/or eggs have been described.^15^ ^,^ ^16^ ^,^ ^17^ Studies using traps to monitor *Aedes* populations in several Brazilian cities have revealed high levels of infestation in urban spaces, and reproductively active females were present in more than 60% of the properties fitted with either traps for catching egg-laying females or with ovitraps.^18^ A longitudinal study of *Ae. aegypti* and *Ae. albopictus* sympatric populations in seven of the 94 neighbourhoods of Recife city (Brazil), carried out by our group in 2003-2005 based on a network of 465 ovitraps, recorded broad and intensive *Aedes* presence in all neighbourhoods.^18^ The overall infestation index was 98.5% and densities from 100 to 2500 eggs/ovitrap/month were recorded throughout the year, regardless of the different environmental and socio-economic conditions of those neighbourhoods. The present study aimed to investigate the infestation pattern of *Ae. aegypti* and *Ae. albopictus* in the Sítio dos Pintos neighbourhood in 2013, compared with a previous assessment of this area^18^ performed in 2005.

## MATERIALS AND METHODS

*Study area* - Recife, the capital of Pernambuco State, is a coastal city characterised by a warm and humid climate with an annual mean temperature of 25.5ºC with narrow variations, greatly favouring mosquito proliferation throughout the year.^19^ Lymphatic filariasis and dengue fever are both endemic in Recife,^18^ ^,^ ^20^ while Chikungunya and Zika viruses were introduced in 2014-2015.^12^ Sítio dos Pintos, one of the 94 neighbourhoods of Recife city, is a suburban area located in the far west of the municipality (8 º 03’S 34 º52’W) adjoining a remnant of the Atlantic Forest. It has a low population density, 7276 inhabitants in 1.8 km^2^ (40,49 people/km²), compared with other neighbourhoods of the city whose average is 142,84 people/km².^21^ In 2005, Recife city recorded 2950 confirmed cases of dengue, while in 2013, only 987 cases were registered. Therefore, Sítio dos Pintos was selected considering the following criteria: it is an urban area located in a transition zone, where *Ae. albopictus* was first recorded in Recife city in 2000;^22^ a subsequent study showed the coexistence of *Ae. aegypti* e *Ae. albopictus* ^18^ and, as described, arboviruses have also been historically reported in Sítio dos Pintos. The incidence coefficient of dengue in this neighborhood was 0.2 in both years. (http://portalsaude.saude.gov.br. Retrieved 07-05-2019).

*Ovitrap surveillance network* - *Ae. aegypi* and *Ae. albopictus* populations from Sítio dos Pintos were assessed for 12 months in 2013, and the data were then compared with partially published results from this same location in 2005.^18^ These periods are respectively named T2 and T1 in this study. The same approach for monitoring *Aedes* populations in T1, which was based on the collection of eggs in a network of 59 georeferenced sentinel ovitraps (s-ovt) treated with *Bacillus thuringiensis sorovar israelensis*-Bti larvicide,^18^ was used in T2. The network from T1 was composed of 87 s-ovt,^18^ that was set in two neighbourhoods, Sítio dos Pintos and Dois Irmãos, and for this study a subset of 59 s-ovt from Sítio dos Pintos was analysed and used to obtain data during T2.

*Ovitrap management* - A modified model of a previously described ovitrap^19^ was used for sampling *Aedes* eggs. These s-ovt were designed to remain safe and functional in the field for collecting eggs for a 30-day period.^18^ Ovitraps were set outdoor, one meter above the soil, in places protected from sun exposure and rainfall. They consist of a black plastic cups filled with water (1.5 L) and two wooden paddles (5 x 15 cm), used as oviposition substrates, vertically fixed to the inner wall of the cup. All s-ovts used to monitoring eggs density were treated with a commercial Bti-based larvicide (VectoBac^®^ WG, 6 drops/trap or ~4.8 mg/L), to prevent larvae development, and protect the ovitraps to become a breeding site, since they were set for a 30-day period. The s-ovt were set in January 2013 and kept continuously for 12 months. Monthly, the s-ovt were inspected to replace water, add Bti and news paddles, as performed in T1.^18^ Labelled paddles collected every month from the s-ovt were transferred to the laboratory and air-dried at room temperature (26ºC). The egg number was recorded under stereoscopic microscope based on all eggs from the paddle, including hatched and non-hatched ones.

*Aedes species identification* - With the purpose to investigate the presence and relative abundance of *Ae. aegypti* and *Ae. albopictus* in the study area, egg samples from 15 s-ovt were analysed twice a year in T1 (May and December 2005) and from 59 s-ovts during T2 (February and December 2013). For this specific investigation, the s-ovt were set without Bti treatment and, after seven days, the paddles were recovered and sent to the laboratory for egg counting. Subsequently the paddles were immersed in water containing grass infusion (5%) to stimulate hatching of first instar larvae (L1) and then transferred to plastic trays with water (4 L) and cat food (Friskies^®^). Those larvae were reared to adult stage for species taxonomic classification. Larvae from each s-ovt were placed in one identified tray to record the distribution of the identified species.^23^ This procedure was repeated for two consecutive weeks for each month/year, after that the s-ovts were treated with Bti, as performed during the whole study.

*Climatic data* - Historical climatic data for Recife, according to the National Institute of Meteorology (INMET),^24^ show an average annual rainfall of 2200 mm³ which is more frequent and intense between February and August. During T1, the rainfall was 2316 mm³ with monthly variations from 8.7 to 709 mm³, the mean monthly temperature varied from 21ºC to 32ºC, and the relative air humidity varied from 70 to 87%. T2 showed total annual rainfall of 2450 mm³ varying from 47.7 to 491.4 mm³ from month to month while the mean monthly temperatures and relative air humidity were similar to those of T1.

*Statistical analysis* - The comparative analysis of the total egg count distribution for each period was performed using box plots, and the significance between these totals was calculated using the Wilcoxon test. The monthly distribution of egg averages by sampling station for each year was also plotted. A spatial smooth kernel density estimator (KDE)^25^ was used to produce surface maps of eggs collected in the s-ovt network over successive counting cycles to identify hot spots of *Aedes* density. Although the KDE is a simple method for analysing focal behavioural patterns, it does so by estimating the intensity of the point process throughout the study region. The KDE parameters were a quadratic function of the KDE kernel with a bandwidth of 120 m. The same grid and colour definitions were applied to all cycles to compare results across time. Analyses were performed using TerraView (www.dpi.inpe.br/terraview), an open Geographic Information System (GIS) application.

*Ethics* - The project was approved by the Research Ethics Committee of the Instituto Aggeu Magalhães (IAM-Fiocruz-PE), Brazil (CAAE: 12640813.7.0000.5190).

## RESULTS

*Infestation by Aedes spp* - In order to evaluate the density and spread of *Aede*s in the study area, a network of 59 s-ovt installed in the same sampling stations during two 12-month cycles corresponding to the years 2005 (T1) and 2013 (T2) was set. The goal of this network of s-ovt was to record egg density for surveillance purpose only, and the same set of 59 s-ovt were used to obtain data from both years analysed. This study did not perform control interventions, and only PNCD actions were carried out by the Health Services in that area, as preconised for all Brazilian municipalities.^9^ In terms of egg density, a total number of 281,103 eggs was collected during T1 which was significantly higher (p < 0.005) than 146,153 eggs recorded for T2 (Table I). The total egg number collected by all 59 s-ovt per month in T1 varied from 8282 to 44,170, while in T2 this ranged from 6792 to 18,174 (Table I). The high infestation pattern found during both periods was characterised by the presence of *Aedes* egg-laying females in all of the sampling stations throughout the year, since the monthly ovitrap positivity index (OPI) varied from 94.3% to 100% (Table I). In terms of eggs abundance, each s-ovt collected from 349 to 20,164 eggs in T1, and during T2 each s-ovt recorded between 178 and 5572 eggs, which also corroborate the sustained infestation over the area [Supplementary data **(Table I)**].

**TABLE I:** Number of *Aedes* eggs collected in 59 sentinel ovitraps (s-ovt) in sampling stations from Sítio dos Pintos, Recife city, carried out in 2005 (T1) and 2013 (T2)

Year	Months											
	1	2	3	4	5	6	7	8	9	10	11	12
T1												
No. eggs (total = 281,103)	9011	9442	8282	25,225	39,926	31,993	44,170	38,835	21,025	22,379	15,268	15,547
Mean/s-ovt^*a*^	158.1	168.6	150.6	450.4	753.3	592.5	883.4	693.5	389.4	414.4	272.6	293.3
Standard deviation	129.9	127.9	125.2	393.3	664.3	561.7	641.5	665.8	437.4	458.8	273.3	308.8
Median	136	154	117	361	537	456	752.5	503	259.5	316	217	207
OPI^*b*^	100.0	94.6	94.5	98.2	100.0	100.0	100.0	100.0	100.0	100.0	96.4	94.3
T2												
No. Eggs (total = 146,153)	13,383	18,174	17,447	15,228	13,105	11,132	10,800	12,289	8078	6792	8485	11,240
Mean/s-ovt	226.8	308.0	295.7	262.6	222.1	188.7	183.1	208.3	136.9	117.1	143.8	193.8
Standard deviation	179.7	233.2	231.1	184.0	147.6	123.6	133.5	201.3	134.3	110.6	116.0	169.9
Median	230	271	244	255	198	157	154	133	98	87.5	116	138
OPI	100.0	96.6	98.3	98.3	100.0	100.0	100.0	96.6	98.3	98.3	98.3	100.0

*a*: in some months, one to three ovitraps from the set of 59 ovitraps were not recorded due to operational matters; *b*: ovitrap positivity index.

**Fig. 1:**
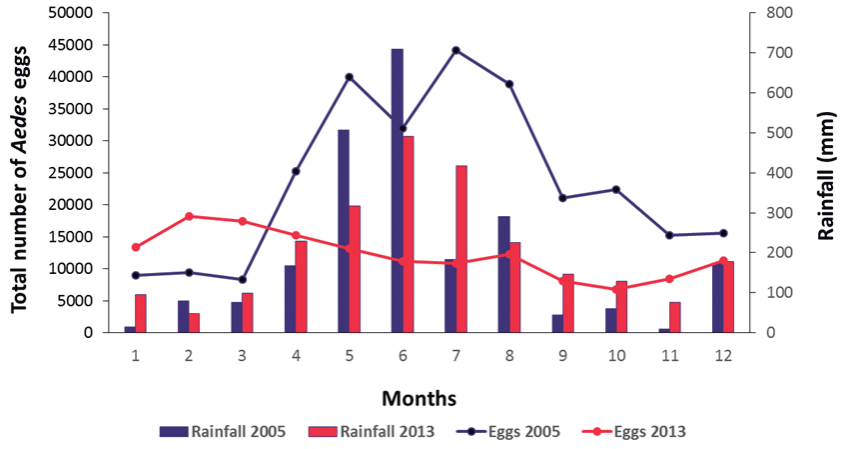
total *Aedes* egg numbers collected by 59 ovitraps in Sítio dos Pintos neighbourhood of Recife-PE from January (1) to December (12) of T1 (2005) and T2 (2013).

The number of eggs recorded for most cycles was lower throughout T2, especially when compared with the densities recorded between May and August in T1, whose peak was detected in July (44.170 eggs) (Table I, Fig. 1). The pluviometric indexes of T1 and T2 presented a similar monthly distribution pattern with peaks between May and August and total amount of rainfall (T1 = 2316 mm³, T2 = 2450 mm³). When the data is analysed throughout the year, a relationship between the rainfall pattern and monthly density of eggs can be observed in T1 (Fig. 1) while in T2, there is no association between these parameters (Fig. 1).

**Fig. 2:**
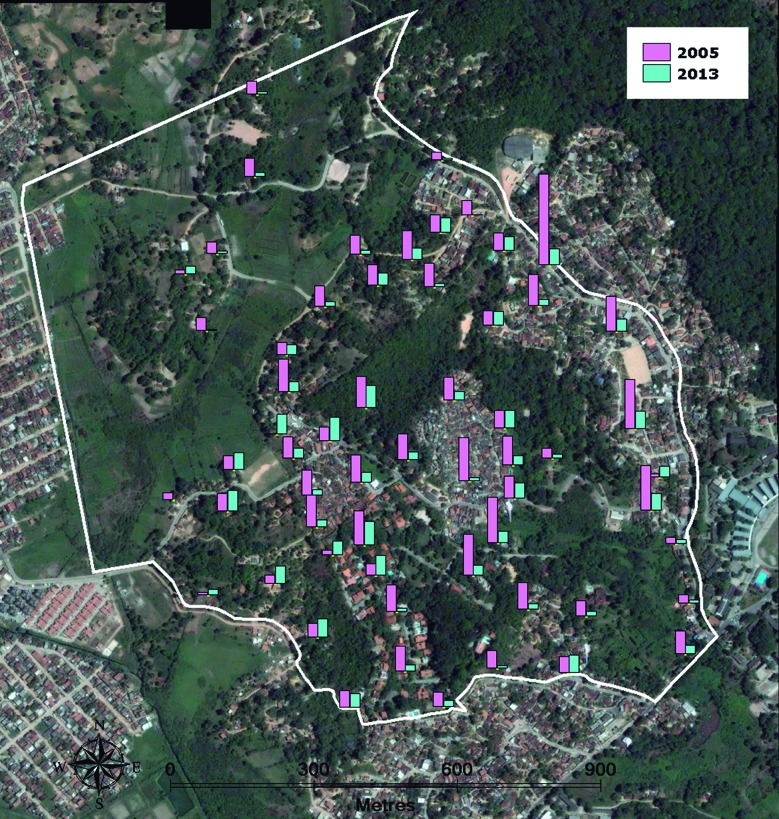
spatial distribution of 59 ovitraps to collect *Aedes* eggs in T1 (2005) and T2 (2013) in Sítio dos Pintos neighbourhood of Recife-PE. Egg numbers collected in the ovitraps are proportionally represented by bars.

**Fig. 3:**
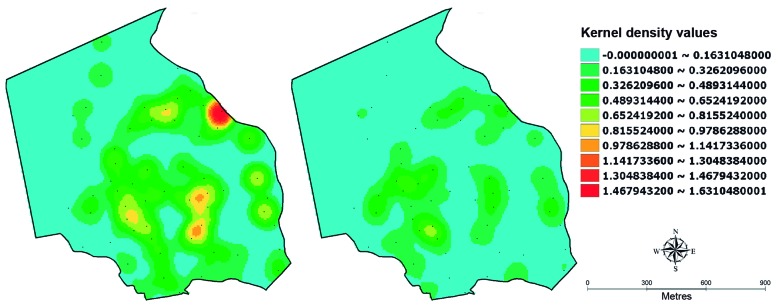
spatial distribution of *Aedes* eggs in Sitio dos Pintos neighbourhood of Recife-PE in T1-2005 (left) and T2-2013 (right) by kernel maps showing smoothed egg densities based on the egg number collected in each of the 59 ovitraps distributed over the urban area during twelve months.

**Fig. 4:**
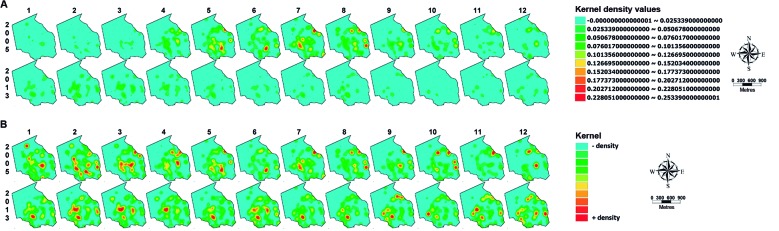
spatial distribution of *Aedes* eggs in Sítio dos Pintos neighbourhood of Recife-PE, during twelve months of years T1 (2005) and T2 (2013). Analysis of the KDEs for each cycle is based on the number of eggs collected in 59 sentinel ovitraps placed over that area. (A) Collective scale maps. (B) Self-scale maps.

The total number of eggs collected per year in each sampling station, when comparing T1 and T2, showed that a decrease in T2, which occurred in most houses monitored (79.7%). In a few stations, no quantitative change occurred, while in 20% of s-ovts, higher egg counts relative to T1 were recorded [Supplementary data **(Table I)**, Fig. 2]. The sampling stations falling into these three categories were not grouped together in the territory but were spatially separate (Fig. 2). KDE maps based on eggs laid on s-ovt showed a heterogeneous spatial distribution over the urban space in T1 as well as in T2 (Fig. 3) but a similar distribution of some hotspots was found whose densities were higher in T1. A comparison of the results across time using KDE maps produced with the same scale for all cycles enables a prompt visual identification of critical periods and areas from month to month, and this can be a valuable tool to establish priority levels for implementing vector control actions. This analysis reveals hotspots with a higher density in T1 throughout the year compared with T2 (Fig. 4A), in particular, between the 5th (May) and 8th (August) cycles, as previously shown in Fig. 1. When these KDE maps are presented in self-scale, which is based on the absolute egg counting from each cycle, areas with a higher egg density are enhanced, in particular in those cycles whose egg density is low (Fig. 4B).

*The spread of Ae. aegypti and Ae. albopictus* - This evaluation for *Ae. aegypti* and *Ae. albopictus* identification was based on a total of 8133 adults obtained from the eggs collected in s-ovt from four assessments, two performed during T1, and two during T2. The overall analysis shows that 70.7% and 29.3% of them were recorded as *Ae. aegypti* and *Ae. albopictus*, respectively (Table II). Both species were found in all four sampling assessments performed for this purpose, with *Ae. aegypti* being predominant (68-85%) in the three first assessments, while the last survey showed a similar abundance (~50%) of both species. Data from this last assessment indicated that fluctuations in the composition of these two species that were collected in the very same stations in this area can take place in short period of time, since there is only a nine month-interval between the assessments (February and December 2013), but the reason behind this change could not be detected.

**TABLE II:** Number of *Aedes aegypti* and *Aedes albopictus* adults identified from eggs collected in sentinel ovitraps (s-ovt) from Sítio dos Pintos neighbourhood of Recife city

Month/Year	No. s-ovt	No. adults	*Ae. aegypti* N (%)	*Ae. albopictus* N (%)
May/2005	15	987	677 (68.6)	310 (31.4)
Dec/2005	15	848	663 (85.2)	185 (14.8)
Feb/2013	59	3878	3129 (81)	749 (19)
Dec/2013	59	2420	1283 (53)	1137 (47)
Total	148	8133	5752 (70.7)	2381 (29.3)

**Fig. 5:**
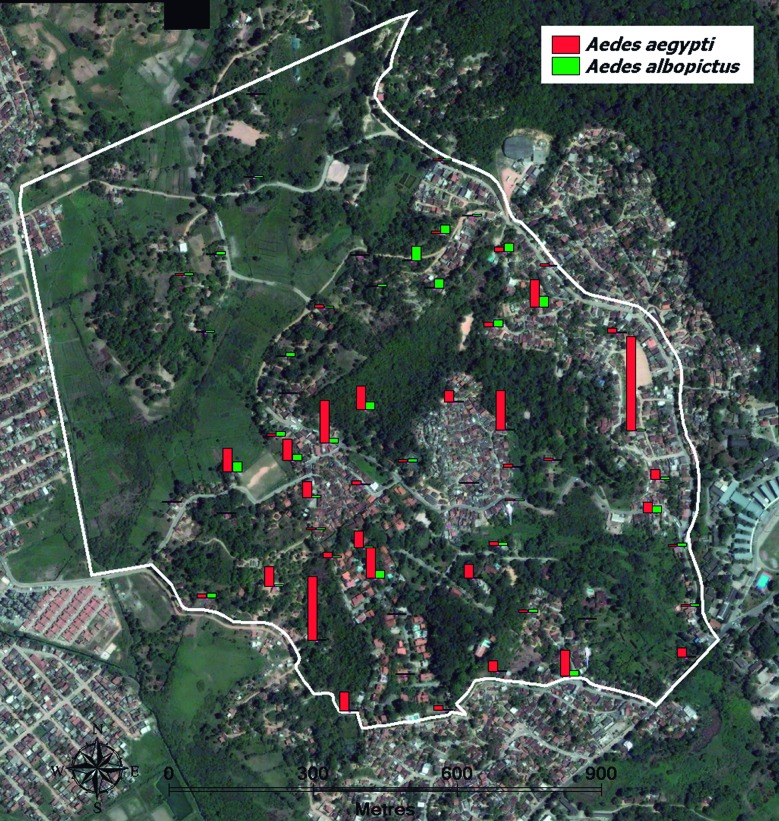
spatial distribution of 59 ovitraps used to collect eggs of *Aedes aegypti* and *Aedes albopictus* in Sítio dos Pintos neighbourhood of Recife-PE throughout the year T2 (2013). The number of individuals from each species is proportionally represented by bars.

The distribution of species considering all egg samples collected in the s-ovt assessed during T1 and T2, shows the simultaneous presence of both species in 65,1% of the traps and the exclusive presence of *Ae. aegypti* or *Ae. albopictus* in 22% and 13,2%, respectively, indicating that these mosquitoes frequently cohabitate the environment in this area and can use the same oviposition sites [Table III, Supplementary data **(Table II)]**. The spread of the two species in Sítio dos Pintos in T2 showed that some s-ovt detected a relative higher abundance of *Ae. albopictus* that were found in houses near areas covered with vegetation (Fig. 5), although fews s-ovt found in such areas showed a higher abundance of *Ae. aegypti*. The results also showed that the mean number of eggs in those stations where *Ae. aegypti* was predominant is much higher than in those where *Ae. albopictus* was found to be the major species [Fig. 5, Supplementary data **(Table II)**]. The spatial distribution of *Aedes* species found in T2 was not compared to those found in T1, as the number of s-ovt used for this purpose in T1 was more limited 15 s-ovt versus 59 s-ovt in T2 and could not provide a comparable dataset.

**TABLE III:** Number of positive sentinel ovitraps (s-ovt) for *Aedes aegypti* and/or *Aedes albopictus* eggs collected in the Sítio dos Pintos neighbourhood of Recife city

	Total	No. of positive s-ovt		Both species
		*Ae. aegypti*	*Ae. albopictus*		
May/2005	10	2	1	7
Dec/2005	9	5	2	2
Feb/2013	58	9	4	45
Dec/2013	52	12	10	30
Total	129	28 (22%)	17 (13.2%)	84 (65.1%)

## DISCUSSION

The pattern of *Aedes* infestation in Sítio dos Pintos neighbourhood from Recife city was assessed during two independent 12-month periods (2005-T1, 2013-T2) and showed major similar features: (i) the detection of egg-laying females in most houses (OPI > 90%); (ii) the presence of a reproductively active population in all months of the year and (iii) the heterogeneous distribution of the population density in all months, but with some sub-areas of persistently higher concentrations. Although the size of the mosquito population in T2, as recorded by the total number of eggs from the ovitrap network, was approximately half that in T1, the ovitrap positivity index continue to be near 100% (96.6-100), which corroborate the permanent occupation of this territory by *Aedes* populations. In addition egg abundance, and density recorded throughout T2 are expressive and corroborates the maintenance of a high infestation level seem in T1.

The monitoring of the *Aedes* population carried out in T1 was part of a broader study conducted in six neighbourhoods of Recife, representing distinct geographical situations and scenarios of urban occupation.^18^ In these areas previously assessed, the characteristics of *Aedes* infestation were similar to those from the present study, despite the substantial environmental and socio-economic differences among the neighbourhoods surveyed.^18^ Sítio dos Pintos, in particular, showed a geographical infestation pattern that showed only discrete alterations in both assessments conducted in T1 and T2, as seem by the data collected by the same ovitrap network used to investigate those periods. Longitudinal studies using this ovitrap network, named “Sistema de Monitoramento e Controle Populacional de *Aedes*” (SMCP-*Aedes*), in other municipalities from Brazil also showed a pattern of permanence of spatial occupation by *Aedes* populations. This pattern was found at urban sites with mosquito populations of contrasting densities such as in Fernando de Noronha Island a low density site^26^ and in the heavily infested neighbourhoods of Brasília Teimosa and Engenho do Meio, from Recife city.^18^

Another important aspect to raise is that the permanent and high level of infestation of *Aedes* in such territories, as Sítio dos Pintos, was detected over almost a decade of systematic municipal control programme actions in the scope of the PNCD. This program preconises six cycles of larvicide application per year, in addition to mechanical actions to eliminate breeding sites in the households.^9^ ^,^ ^10^ Although the total number of eggs recorded in Sítio dos Pintos in T2 was lower compared to T1, it is not possible to conclude if this was only a consequence of the PNCD actions and/or from other factors as, for instance, natural fluctuations of population density that could occur throughout the years. Longitudinal entomological studies could clarify the amplitude of such variations throughout the years, however, those are not available for this study area.

Therefore the results from the present study corroborate previous data from Santa Cruz do Capibaribe city (SCC), a municipality from Pernambuco State in Brazil,^19^ which indicated that *Aedes* populations can be well established in urban areas, despite the PNCD actions. In SCC, for instance, a continuous network of s-ovt recorded a widespread *Ae. aegypti* infestation with a 97-100% trap positivity and an estimated average of 1587 (67-6027) eggs/trap/month in 2008-2009,^19^ after one decade of PNCD control actions in this city. On the other hand, after a two-year trial that employed additional intensive control interventions targeting eggs, larvae and adults, a significant level of control with a 90% decrease in the egg density was finally achieved in that city.^19^ This trial proved that a marked reduction in the *Aedes* population could be achieved when suitable, intensive and sustained control measures were adopted and maintained in order to result in the significant mass elimination of eggs/larvae detected in SCC city. These results provide practical evidence of Schofield’s claim^19^ regarding the need for intensive control pressure maintained long enough to impact a population of vector mosquitoes.

In this study the monitoring system based in the s-ovt network characterised the occupation pattern of *Aedes* due to its sensibility to detect active females in the areas and to provide a quantitative dataset of eggs sampling, and corroborates previous studies carried in endemic areas from Brazil.^18^ Dataset from our study demonstrated that the House index (HI), the indicator used by the PNCD, did not reflect the infestation of Sítio dos Pintos by *Aedes* in 2005 or in 2013, as the HI was < 1 (http://portalsaude.saude.gov.br. Retrieved 07-05-2019), while the OPI shown by our study was close to 100%. This monitoring method based on the visual identification of larvae/pupae has been used for three decades in Brazil, although its lack of efficacy has been shown by studies carried out in other cities.^26^ ^,^ ^27^ In addition, these previous studies have also demonstrated the effectiveness of a modified ovitrap network for controlling *Aedes* population density in urban areas, targeting the mass egg removal by ovitraps, as described for the control program established in SCC city.^19^ Our study showed that the network of s-ovt associated to the KDE maps, for instance, are strategic and simple methods to identify hot spots of *Aedes* density that can be used to follow the spatial-temporal patterns of density in areas, and establish priorities for control actions. It is important that the output of data analysis should be readily readable and understood by health staff at all levels, as suggested by previous studies.^18^

The infestation pattern of *Aedes* population should also be analysed in the light of the arbovirus transmission dataset. According to the epidemiological bulletins of Recife, arbovirus cases in Sítio dos Pintos residents have been confirmed in 10 of the 15 years between 2005 and 2014 (excepting 2007 and 2009) and the greatest number of cases reported were in 2005 (10), 2006 (12), 2008 (42), 2010 (3), 2011 (1), 2012 (8), 2013 (10), 2014 (9) and 2015 (3). This indicates that the decrease in the vector population size observed in 2013 in that area was not enough to prevent arbovirus transmission in the same and in the subsequent years, although PNCD control actions continue to be carried out until the present.

Recife had its first dengue epidemic in 1987, and from 1995 to the present, there has been a prolonged period of annual transmission of the virus with severe outbreaks. The introduction of CHIKV in 2014 and ZIKV in 2015, worsened the epidemiological situation and the pattern of mosquito infestation in many areas of Recife city, including Sítio dos Pintos, allowing the transmission of arboviruses at important levels. In 2019, up to the 52nd epidemiological week (December) 61.451 dengue, 8.467 Chikungunya and 3.827 Zika notified cases were reported in Recife, and this virus transmission has always been attributed to *Ae. aegypti*,^28^ although *Culex quinquefasciatus* females have been collected naturally infected with ZIKV.^29^

Besides that, the presence of *Ae. albopictus* in Brazil was first recorded in 1986 in Rio de Janeiro,^30^ while in Recife it was detected only after 1996, within PNCD larval surveillance. Its first notification in a silvatic area was in a remnant of the Atlantic Forest^22^ closely located to Sítio dos Pintos, our study area. The present study showed a widespread population of *Ae. albopictus*, in this neighbourhood whose eggs were found in 67% of s-ovts in T1 and 75% in T2. In those same samples, most s-ovts were colonised by both species demonstrating their co-occupation of the environment and the potential colonisation of the same oviposition sites in that area. Moreover, no difference was observed in the relative abundance of *Aedes* species in the samples collected eight years later, when ~70% of the eggs were laid by *Ae. aegypti* and ~30% by *Ae. albopictus*. Dataset showed that *Ae. aegypti* and *Ae. albopictus* coexist in the same physical space in an area of ongoing human population expansion with vegetation spots that displays both urban and rural characteristics, and our dataset from two independent moments do not provide evidence of the displacement of *Ae. aegypti* by *Ae. albopictus*. Therefore, their populations are not concentrated in clearly distinct territories, as it has been observed in other studies which showed *Ae. albopictus* was associated with more vegetation and *Ae. aegypti* in urban sprawl,^28^ ^,^ ^30^ and Sítio dos Pintos area could be under transition. The high percentage of traps simultaneously containing eggs of both species appears to indicate a process of continuous dispersion and naturally occupying the breeding sites available in the environment, as expected from invasive species.

*In conclusion* - Populations of *Ae. aegypti* and *Ae. albopictus* have been stably established in the Sítio dos Pintos neighbourhood whose occupation in the urban space is characterised by the continuous presence of a reproductively active vector population in most houses throughout two years analysed. Vector control actions performed in the scope of the National Program of Dengue Control have not a substantial impact in the pattern of infestation found in the years of 2005 and 2013 and, although the absolute number of eggs recorded in 2013 was lower, *Aedes* infestation is still high and well spread. The present study demonstrated the robustness of the spatial occupation and permanence of *Ae. aegypti* and *Ae. albopictus* populations in endemic urban areas, indicates the need to adopt complementary methodologies to improve the surveillance and control of these species.

## References

[1] Bhatt S, Gething PW, Brady OJ, Messina JP, Farlow AW, Moyes CL (2013). The global distribution and burden of dengue. Nature.

[2] Kraemer MUG, Sinka ME, Duda KA, Mylne AQN, Shearer FM, Barker CM (2015). The global distribution of the arbovirus vectors Aedes aegypti and Ae albopictus. ELife.

[3] Patterson J, Sammon M, Garg M (2016). Dengue, Zika and Chikungunya emerging arboviruses in the New World. West J Emerg Med.

[4] Powell JR, Tabachnick WJ (2013). History of domestication and spread of Aedes aegypti ? A Review. Mem Inst Oswaldo Cruz.

[5] Suter T, Crespo MM, Oliveira MF, Oliveira TSA, Melo-Santos MAV, Oliveira CMF (2017). Insecticide susceptibility of Aedes albopictus and Ae aegypti from Brazil and the Swiss-Italian border region. Parasit Vectors.

[6] Carvalho RG, Lourenço-de-Oliveira R, Braga IA (2014). Updating the geographical distribution and frequency of Aedes albopictus in Brazil with remarks regarding its range in the Americas. Mem Inst Oswaldo Cruz.

[7] Martins VEP, Alencar CH, Kamimura MT, Araujo FMC, Simone SG, Dutra RF (2012). Occurrence of natural vertical transmission of dengue-2 and dengue-3 viruses in Aedes aegypti and Aedes albopictus in Fortaleza, Ceará, Brazil. PLoS One.

[8] Gubler DJ (2011). Dengue, urbanization and globalization the unholy trinity of the 21(st) Century. Trop Med Health.

[9] MS - Ministério da Saúde (2002). Programa Nacional de Controle da Dengue (PNCD).

[10] Ministério da Saúde (2009). Secretaria de Vigilância em Saúde. Departamento de Vigilância Epidemiológica. Diretrizes nacionais para a prevenção e controle de epidemias de dengue. Ministério da Saúde.

[11] Teixeira MG, Siqueira JB, Ferreira GLC, Bricks L, Joint G (2013). Epidemiological trends of dengue disease in Brazil (2000-2010) a systematic literature search and analysis. PLoS Negl Trop Dis.

[12] Zanluca C, Andrade de Melo VC.Mosimann ALP.dos Santos GIV.dos Santos CND.Luz K (2015). First report of autochthonous transmission of Zika virus in Brazil. Mem Inst Oswaldo Cruz.

[13] Codeço CT, Lima AWS, Araújo SC, Lima JBP, Maciel-de-Freitas R, Honório NA (2015). Surveillance of Aedes aegypti comparison of House Index with four alternative traps. PLoS Negl Trop Dis.

[14] Morato VC, Teixeira MG, Gomes AC, Bergamaschi DP, Barreto ML (2005). Infestation of Aedes aegypti estimated by oviposition traps in Brazil. Rev Saude Publica.

[15] Santos SR, Melo-Santos MAV, Regis LN, Albuquerque CMR (2003). Field evaluation of ovitraps consociated with grass infusion and Bacillus thuringiensis var israelensis to determine oviposition rates of Aedes aegypti. Dengue Bull.

[16] Facchinelli L, Valerio L, Pombi M, Reiter P, Costantini C, Torre D (2007). Development of a novel sticky trap for container-breeding mosquitoes and evaluation of its sampling properties to monitor urban populations of Aedes albopictus. Med Vet Entomol.

[17] Gama RA, Silva E, Silva I, Resende MC, Eiras AE (2007). Evaluation of the sticky MosquiTRAPTM for detecting Aedes (Stegomyia) aegypti (L ) (Diptera: Culicidae) during the dry season in Belo Horizonte, Minas Gerais, Brazil. Neotrop Entomol.

[18] Regis LN, Monteiro AM, Melo-Santos MAV, Silveira JC, Furtado AF, Acioli RV (2008). Developing new approaches for detecting and preventing Aedes aegypti population outbreaks basis for surveillance, alert and control system. Mem Inst Oswaldo Cruz.

[19] Regis LN, Acioli RV, Silveira JC, Melo-Santos MAV, Souza WV, Ribeiro CNM (2013). Sustained reduction of the dengue vector population resulting from an integrated control strategy applied in two Brazilian cities. PLoS One.

[20] Regis L, Silva-Filha MHNL, de Oliveira CMF, Rios EM, da Silva SB, Furtado AF (1995). Integrated control measures against Culex quinquefasciatus, the vector of filariasis in Recife. Mem Inst Oswaldo Cruz.

[21] Instituto Brasileiro de Geografia e Estatística (2010). http://www.ibge.gov.br/censo2010.

[22] Albuquerque CMR, Melo-Santos MAV, Bezerra MAS, Barbosa RMR, Silva DF, Silva E (2000). Primeiro registro de Aedes albopictus em área da Mata Atlântica, Recife, PE, Brasil. Rev Saude Publica.

[23] Forattini OP (1965). Entomologia Médica. Culicini: Culex, Aedes e Psorophora. Vol. II. Editora da USP.

[24] Instituto Nacional de Meteorologia (2013). http://www.inmet.gov.br.

[25] Bailey TC, Gatrell AC (1995). Interactive spatial data analysis.

[26] Regis LN, Acioli RV, Silveira JC, de Melo-Santos MAV, da Cunha MC, Souza F (2014). Characterization of the spatial and temporal dynamics of the dengue vector population established in urban areas of Fernando de Noronha, a Brazilian oceanic island. Acta Trop.

[27] Maciel-de-Freitas R, Neto RB, Gonçalves JM, Codeço CT, Lourenço-de-Oliveira R. (2006). Movement of dengue vectors between the human modified environment and an urban forest in Rio de Janeiro. J Med Entomol.

[28] Braks MAH, Honório NA, Lourenço-de-Oliveira R, Juliano SA, Lounibos LP (2003). Convergent habitat segregation of Aedes aegypti and Aedes albopictus (Diptera Culicidae) in southeastern Brazil and Florida. J Med Entomol.

[29] Ayres CFJ, Guedes DRD, Paiva MHS, Morais-Sobral MC, Krokovsky L, Machado LC (2019). Zika virus detection, isolation and genome sequencing through Culicidae sampling during the epidemic in Vitória, Espírito Santo, Brazil. Parasit Vectors.

[30] Pancetti FGM, Honório NA, Urbinatti PR, Lima-Camara TN (2015). Twenty-eight years of Aedes albopictus in Brazil a rationale to maintain active entomological and epidemiological surveillance. Rev Soc Bras Med Trop.

