# Supplementary material for: Infestation of an endemic arbovirus area by sympatric populations of Aedes aegypti and Aedes albopictus in Brazil
Source: Mem Inst Oswaldo Cruz. 2020 May 18;115:e190437. doi: 10.1590/0074-02760190437 (PMC7233267; doi:10.1590/0074-02760190437)
Supplement: Supplementary file 1 [file 1678-8060-mioc-115-e190437-s.pdf]

TABLE I

Total number of *Aedes* eggs collected during twelve months from 2005 (T1) and 2013 (T2) in 59 sentinel ovitraps (s-ovt) set in Sítio dos Pintos area

Station	T1	T2	Station	T1	T2
1	1868	178	31	5304	2258
2	9837	1093	32	6724	2946
3	3468	437	33	2152	1017
4	3188	522	34	3479	1738
5	6135	1148	35	5078	2569
6	20,164	3847	36	2539	1286
7	5547	1066	37	4875	3068
8	4224	979	38	5255	3657
9	7227	1688	39	7854	5538
10	6248	1505	40	7234	5229
11	7390	1826	41	4258	3336
12	3295	888	42	1675	1326
13	9227	2523	43	4112	3344
14	10,297	2838	44	3069	2565
15	5875	1653	45	4150	3473
16	5881	1683	46	3529	3309
17	4318	1239	47	4205	4167
18	4497	1291	48	3866	4136
19	4798	1421	49	4226	4810
20	7537	2537	50	3386	3964
21	2814	963	51	3300	4365
22	6641	2330	52	3424	5572
23	6081	2139	53	1254	2046
24	2135	772	54	2856	4707
25	11,060	4136	55	887	1652
26	8026	3010	56	2220	4272
27	3792	1423	57	1469	3322
28	10,099	4024	58	1132	2802
29	6312	2535	59	349	4633
30	5291	2241	Total	281.103	146.153

TABLE II

Number of *Aedes aegypti* and *Aedes albopictus* adults identified from eggs collected in sentinel ovitraps (s-ovt) in the Sítio dos Pintos neighbourhood of Recife-PE

	February/2013				December/2013			
	Ae. aegypti		*Ae. albopictus*		*Ae. aegypti*		*Ae. albopictus*	
Station	Male	Female	Male	Female	Male	Female	Male	Female
116	0	2	1	3	0	0	1	2
412	3	4	22	18	2	0	39	38
207	1	0	25	20	0	0	0	0
411	1	0	10	0	0	0	107	84
406	2	1	2	1	0	0	2	2
115	0	0	3	2	1	0	12	29
301	1	0	0	0	0	0	0	0
204	0	0	1	2	0	0	0	12
118	1	0	6	7	0	1	33	32
205	1	2	6	2	0	0	0	0
320	1	2	7	3	15	23	1	3
117	0	0	2	0	0	0	2	2
305	14	6	2	0	10	4	2	4
208	56	52	21	7	5	5	0	0
312	8	10	1	1	3	1	1	0
121	3	5	7	4	0	1	3	5
109	2	0	0	0	2	1	0	0
303	31	28	0	1	1	0	0	0
310	94	112	2	0	8	9	0	0
415	13	0	0	0	1	0	0	0
214	67	75	16	37	0	0	1	0
211	7	12	25	9	0	0	30	39
404	10	10	30	12	2	1	31	27
413	6	2	0	0	68	25	56	83
110	255	244	2	1	8	9	8	1
418	47	3	9	0	21	19	1	3
317	0	4	7	6	0	0	1	0
220	30	21	23	11	80	91	17	14
420	3	0	4	0	95	87	33	18
315	17	10	0	2	3	4	4	4
123	6	20	0	5	0	4	6	5
122	8	2	11	1	1	1	0	0
125	24	29	0	0	1	2	0	0
225	39	30	0	0	32	15	4	0
323	53	44	1	1	11	3	1	0
421	2	5	0	1	4	3	1	4
224	6	10	17	2	1	5	0	0
321	0	0	0	0	0	0	0	0
401	1	0	0	0	0	2	0	0
424	54	46	0	0	41	27	1	0
325	38	96	11	14	0	0	0	0
422	56	44	4	0	118	117	6	19
316	35	51	2	2	28	29	2	2
324	15	5	0	2	1	1	0	0
425	27	19	1	1	15	19	0	0
124	16	0	11	1	14	17	0	0
217	168	169	0	2	57	29	8	0
319	73	84	7	29	0	0	0	0
Total	1536	1593	395	354	696	587	553	584

CC BY
